# Afferent vagal nerve stimulation resets baroreflex neural arc and inhibits sympathetic nerve activity

**DOI:** 10.14814/phy2.12136

**Published:** 2014-09-04

**Authors:** Keita Saku, Takuya Kishi, Kazuo Sakamoto, Kazuya Hosokawa, Takafumi Sakamoto, Yoshinori Murayama, Takamori Kakino, Masataka Ikeda, Tomomi Ide, Kenji Sunagawa

**Affiliations:** 1 Department of Cardiovascular Medicine, Kyushu University Graduate School of Medical Sciences, Fukuoka, Japan; 2 Department of Advanced Therapeutics for Cardiovascular Diseases, Kyushu University Graduate School of Medical Sciences, Fukuoka, Japan

**Keywords:** Afferent nerve, carotid sinus baroreflex, sympathetic nerve activity, vagal nerve stimulation

## Abstract

It has been established that vagal nerve stimulation (VNS) benefits patients and/or animals with heart failure. However, the impact of VNS on sympathetic nerve activity (SNA) remains unknown. In this study, we investigated how vagal afferent stimulation (AVNS) impacts baroreflex control of SNA. In 12 anesthetized Sprague–Dawley rats, we controlled the pressure in isolated bilateral carotid sinuses (CSP), and measured splanchnic SNA and arterial pressure (AP). Under a constant CSP, increasing the voltage of AVNS dose dependently decreased SNA and AP. The averaged maximal inhibition of SNA was ‐28.0 ± 10.3%. To evaluate the dynamic impacts of AVNS on SNA, we performed random AVNS using binary white noise sequences, and identified the transfer function from AVNS to SNA and that from SNA to AP. We also identified transfer functions of the native baroreflex from CSP to SNA (neural arc) and from SNA to AP (peripheral arc). The transfer function from AVNS to SNA strikingly resembled the baroreflex neural arc and the transfer functions of SNA to AP were indistinguishable whether we perturbed ANVS or CSP, indicating that they likely share common central and peripheral neural mechanisms. To examine the impact of AVNS on baroreflex, we changed CSP stepwise and measured SNA and AP responses with or without AVNS. AVNS resets the sigmoidal neural arc downward, but did not affect the linear peripheral arc. In conclusion, AVNS resets the baroreflex neural arc and induces sympathoinhibition in the same manner as the control of SNA and AP by the native baroreflex.

## Abstract (precis)

Afferent vagal nerve stimulation resets the baroreflex neural arc and inhibits sympathetic nerve activity.

## Introduction

Autonomic imbalance characterized by sustained excessive sympathetic excitation and parasympathetic withdrawal plays a pivotal role in aggravation of chronic heart failure (CHF) (Schwartz et al. 1998; Schwaltz and De Ferrari 2011). Sympathovagal imbalance in CHF patients may increase heart rate, oxygen consumption, arrhythmias, and mortality rate (Sabbah et al. 1994; La Rovere et al. 1998; Lechat et al. 2001). Based on these findings, electrical vagal nerve stimulation (VNS) for the treatment of CHF has been developed and shown to prevent sudden cardiac death in dogs with myocardial infarction (Vanoli et al. 1991) and improve long‐term survival in rats with CHF (Li et al. 2004). Recently, De Ferrari et al. (De Ferrari et al. 2011) reported the safety and tolerability of a VNS system (CardioFit; BioControl, Yehud, Israel) in CHF patients and also showed significant improvements of exercise tolerance and left ventricular performance. Various possible mechanisms for the beneficial effects of efferent vagal pathway stimulation on heart failure have been proposed, such as lowering of heart rate and oxygen consumption (Shimizu et al. 2009), reduction in inflammation through nicotinic receptors (Tracey 2007), attenuation of norepinephrine spillover into the left ventricle (Levy and Blattberg 1976), and suppression of free radical generation (Tsutsumi et al. 2008). On the other hand, since the vagal afferent fibers are connected to the nucleus tractus solitarius that regulates sympathetic nerve activity (SNA) (Smith et al. 1998), VNS has been suggested to reduce SNA by stimulating vagal afferent fibers. Because the arterial baroreflex operates as a negative‐feedback system and regulates SNA tightly to stabilize arterial pressure (AP), an operation of baroreflex obscures the pure VNS effects on SNA and AP. To elucidate the pure effects of VNS on sympathetic AP regulation at various levels of SNA, a baroreflex open‐loop analysis is required (Kawada et al. 1997; Kamiya et al. 2011).

The sympathetic arterial baroreflex can be divided into two subsystems; a neural arc that describes how the baroreceptor pressure changes SNA, and a peripheral arc that describes how the SNA changes AP (Sato et al. 1999). Kawada et al. (Kawada et al. 2011) reported that selective efferent vagal stimulation does not change either the neural or the peripheral arc. Kashihara et al. 2003 reported that activation of vagal afferent pathway by phenylbiguanide (Bezold–Jarisch reflex) suppresses sympathoexitation and attenuates baroreflex dynamic gain. However, how selective vagal afferent stimulation (AVNS) modifies SNA and baroreflex function has not been fully elucidated. With this background, we investigated the isolated effect of AVNS on baroreflex control of SNA and AP regulation.

## Materials and Methods

### Animals and surgical preparations

Experiments and animal care were approved by the Committee on Ethics of Animal Experiment, Kyushu University Graduate School of Medical Sciences, and performed in strict accordance with the Guide for the Care and Use of Laboratory Animals published by the US National Institutes of Health. Twelve male Sprague–Dawley rats (646 ± 25 g) were anesthetized by an intraperitoneal injection (2 mL/kg) of a mixture of *α*‐chloralose (40 mg/mL) and urethane (250 mg/mL), and ventilated mechanically with oxygen‐enriched gas. The depth of anesthesia was maintained with a 20‐fold diluted solution of the above anesthetic mixture infused from the right femoral vein (2–3 mL/kg/h). AP was measured using a high‐fidelity pressure transducer (SPR–320; Millar Instruments, Houston, TX) inserted into the right femoral artery. Body temperature was maintained by a heating pad at approximately 38°C. A postganglionic branch of the splanchnic sympathetic nerve was exposed through a left flank incision. A pair of stainless steel wire electrodes (Bioflex wire AS633; Cooner Wire, Chatsworth, CA) was attached to the nerve to record SNA. The nerve and electrodes were secured and insulated with silicone glue (Kwik‐Sil; World Precision Instruments, Sarasota, FL). To quantify SNA, a preamplified nerve signal was band‐pass filtered at 150–1000 Hz, and then full‐wave rectified and low‐pass filtered at a cutoff frequency of 30 Hz using analog circuits. Pancuronium bromide (0.4 mg/kg/h) was infused continuously to prevent electrical contamination of SNA resulting from muscular activity. At the end of the experiment, a bolus injection of a ganglionic blocker hexamethonium bromide (60 mg/kg) was given to confirm the disappearance of SNA and to measure the noise level. Carotid sinus baroreceptor regions were isolated from the systemic circulation according to previously reported procedures (Sato et al. 1999) with modifications. Briefly, a 5–0 silk thread was passed between the external and internal carotid arteries, and the external carotid artery was ligated close to the carotid bifurcation. The internal carotid artery was embolized with three to five steel balls (0.8 mm steel ball; Tsubaki Nakashima, Nara, Japan) injected from the common carotid artery. The isolated carotid sinuses were filled with saline through catheters inserted into the common carotid arteries. Carotid sinus pressure (CSP) was controlled using a servo‐controlled piston pump. Heparin sodium (100 U/kg) was injected intravenously to prevent blood coagulation. Bilateral aortic depressor nerves were sectioned at the neck to avoid reflexes from the aortic arch.

#### Afferent vagal nerve stimulation

The right vagus nerve was sectioned at the neck position, and a pair of stainless steel wire electrodes (Bioflex wire AS633; Cooner Wire, Chatsworth, CA) was attached to the sectioned central end of the right vagus for AVNS. The left vagal nerve was cut at the neck level. The nerve and electrodes were secured and insulated with silicone glue. AVNS was performed using an electric rectangular wave current with a fixed frequency of 20 Hz and pulse duration of 0.2 msec, which was used for chronic VNS in a previous rat study (Li et al. 2004). The amplitude of electrical stimulation was changed in each protocol.

### Protocols

After the surgical procedures were completed, responses of SNA and AP to CSP input were monitored for more than 30 min. The rat was excluded from the study if the reflex responses diminished during this stabilization period. After stabilization, CSP was matched to AP to close the carotid sinus baroreflex, using a high performance servo‐controlled piston pump that was able to reproduce the instantaneous pulsatile AP in the carotid sinus (Sato et al. 1999), and baseline hemodynamic data (operating point) were recorded for 10 min. Six of 12 rats were allocated to protocols 1 and 2, and the other 6 rats were allocated to protocol 3.

#### Protocol 1: Static effects of AVNS on SNA and AP

To assess the pure relationship between AVNS and SNA, we maintained a constant CSP (pressure at operating point) to avoid the buffering effect through the baroreflex. We administered AVNS for 80 sec and assessed the changes in SNA and AP. The amplitude of AVNS was increased from 0 to 8 V (0, 2, 4, 6, and 8 V).

#### Protocol 2: Dynamic effects of AVNS on SNA and AP

We compared the dynamic responses of SNA and AP as a result of AVNS with those of native arterial baroreflex. To obtain AVNS‐SNA and SNA‐AP data sets, we altered the duty cycle of AVNS every 500 msec according to a random binary sequence, and maintained constant CSP (pressure at operating point) to avoid the buffering effect through the baroreflex in the AVNS study. To obtain dynamic baroreflex response, we determined mean AP under baseline conditions and used this as operating AP. Then we matched mean CSP to the operating AP and altered CSP by 20 mmHg above or below the operating AP every 500 msec according to a random binary sequence, and obtained the CSP‐SNA and SNA‐AP data sets (Kamiya et al. 2011). These random perturbations were performed for 10 to 20 min, and the data set for a stable 5‐min segment was used for analyses. We identified the transfer functions (H) and compared the transfer functions of ANVS to SNA (H*_AVNS‐SNA_*) and SNA to AP (H*_SNA‐AP_*) with those of baroreflex neural (H*_CSP‐SNA_*) and peripheral (H*_SNA‐AP_*) arcs. The amplitude of AVNS was adjusted to 6–8 V to minimize SNA in each animal.

#### Protocol 3: Effect of AVNS on baroreflex function

To estimate the open‐loop function of the arterial baroreflex, CSP was first decreased to 60 mmHg and then increased stepwise from 60 to 170 mmHg every 20 sec. We compared the responses of SNA and AP to CSP changes in the presence and absence of AVNS. The amplitude of AVNS was adjusted to 6–8 V, which minimized SNA in each animal.

### Data analyses

Experimental data pairs were recorded at a 200‐Hz sampling interval using a 16‐bit analog‐to‐digital converter (Power Lab 16/35; ADInstruments, NSW, Australia) and stored in a dedicated laboratory computer system. In protocol 1, SNA and AP measured before (baseline) and during AVNS were averaged for the last 20 sec in each animal. In protocol 2, the transfer functions were estimated by the following procedure. The input–output data pairs were divided into 5 segments and processed with 50% overlapping bins of 1024 points each by a fast Fourier transform algorithm to identify transfer function. For each segment, the linear trend was subtracted and a Blackman–Harris window was applied. A fast Fourier transform was performed to obtain the spectra of the data segments. The ensemble averages of input [S_*xx*_ (*f*)], output [S_*yy*_ (*f*)], and cross‐spectral signal between input and output [S_*yx*_ (*f*)] were estimated over the eight segments. Finally, the transfer function [*H*(*f*)] from the input to the output was calculated as follows (Kent et al. 1972). 
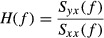


Because the amplitude of AVNS and carotid sinus pressure had different units, we were not able to compare the absolute dynamic gain of the transfer function of AVNS to SNA and CSP to SNA. We normalized the transfer functions using the mean dynamic gain below 0.03 Hz when we compared the transfer function of AVNS to SNA with that of the baroreflex neural arc.

We obtained the gain [*H*(*f*)] and phase [*θ* (*f*)] of the transfer function using the following equations (Kent et al. 1972). 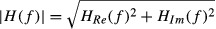


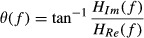


To quantify the linear dependence between the input and output signals in the frequency domain, a magnitude‐squared coherence function [Coh (*f*)] was calculated as follows (Kent et al. 1972). 
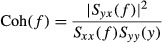


The coherence value ranges from zero to unity. Unity coherence indicates perfect linear dependence between input and output signals, whereas zero coherence indicates total independence between the two signals.

In protocol 3, to estimate the input–output relationship at the steady state, SNA and AP were averaged over the last 10 sec at each CSP level of the stepwise increased input. Static characteristics of the carotid sinus baroreflex neural arc (CSP–SNA relationship) approximate an inverse sigmoid curve, and are quantified using a four‐parameter logistic function as follows (Sato et al. 1999; Kawada et al. 2011). 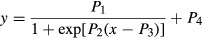


where *x* and *y* represent the input (CSP) and the output (SNA) values, respectively; *P*_1_ is the response range of y; *P*_2_ is the slope coefficient; *P*_3_ is the midpoint of the sigmoid curve on the CSP axis; and *P*_4_ is the minimum value of y. The maximum gain (G max) is ‐(*P*_1_×*P*_2_)/4 at *x* = *P*_3_. Static characteristics of the peripheral arc (SNA–AP relationship) approximate a straight line, and are quantified using a linear regression as follows: 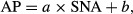
where *a* and *b* represent the slope and intercept, respectively. The obtained SNA were normalized using the average integrated value across the period when CSP = 60 mmHg in protocol 3.

### Statistical analysis

Data are presented as means ± SD in protocols 1 and 3. In protocol 2, data are presented as means ± SEM. Differences were considered significant when *P* <**0.05. In protocol 1, the effects of AVNS on AP and SNA at different time intervals were evaluated by one‐way ANOVA. The Dunnett's test was used for multiple comparisons. In protocol 2, to test the difference between the AVNS and arterial baroreflex conditions, we obtained the gain and phase values at 0.01, 0.1, 0.5, and 0.75 Hz in each animal. The group differences in these values between two conditions were examined by Student's t‐test. In protocol 3, the effects of AVNS on the parameters of the logistic and linear functions related to the neural and peripheral arcs, as well as on the closed‐loop operating point were examined using paired *t*‐test.

## Results

### AVNS impact on sympathetic nerve activity

**Figure 1.**
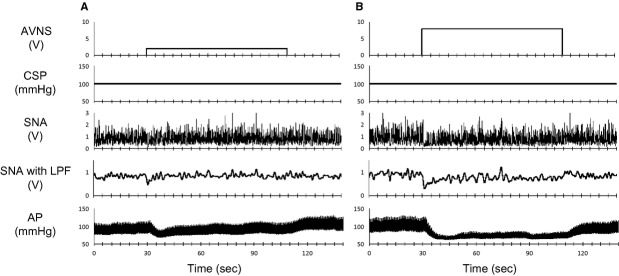
Representative time series of sympathetic nerve activity (SNA) and arterial pressure (AP) during low‐ (A) and high‐amplitude (B) afferent vagal nerve stimulation (AVNS) from one rat. Data include 30 sec of baseline (0–30 sec), 80 sec of AVNS (30–110 sec), and 30 sec of recovery (110–140 sec). CSP, carotid sinus pressure; LPF, low‐pass filter.

**Figure 2.**
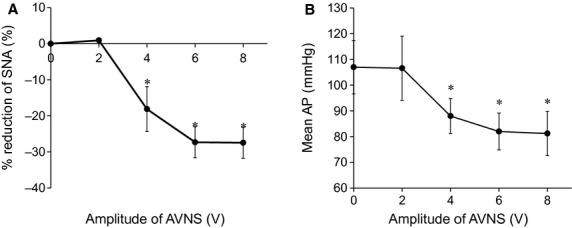
Mean percent changes in sympathetic nerve activity (SNA, A) and mean aortic pressure (Mean AP, B) under afferent vagal nerve stimulation (AVNS) at various amplitudes. Data are expressed as means ± SD (*n* = 6). AVNS at 4, 6, and 8 V significantly inhibited SNA and mean AP. *P <**0.05 versus baseline values (0 V).

Figure 1 shows typical time series of SNA and AP in response to low (2 V, Fig. 1A)‐ and high (8 V, Fig. 1B)‐amplitude AVNS. SNA was reduced immediately after AVNS was started and reached a steady state in 20–30 sec. AP also decreased following the SNA reduction. In low‐amplitude AVNS (2 V), both steady state SNA and AP during AVNS did not differ from baseline. In contrast, in high‐amplitude AVNS (8 V), both steady state SNA and AP during AVNS were significantly suppressed compared to baseline. Figure 2 illustrates the group averaged SNA reduction and mean AP in response to various intensities of AVNS. AVNS above 4 V significantly inhibited SNA and mean AP, demonstrating a voltage threshold and saturation level. The maximum SNA inhibition was ‐28.0 ± 10.3%. Although not shown in Figure 2, the reduced SNA and AP returned to baseline levels after the cessation of AVNS.

### Dynamic AVNS impact on SNA and AP

**Figure 3.**
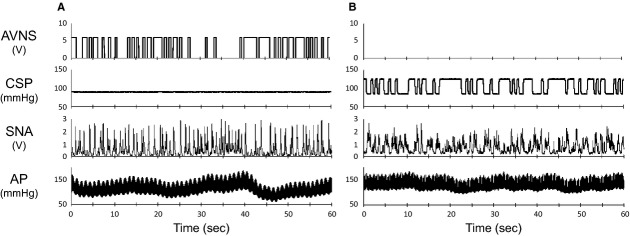
Representative time series of sympathetic nerve activity (SNA) and arterial pressure (AP) under perturbations by afferent vagal nerve stimulation (AVNS) (A) and carotid sinus pressure (CSP) (B) from one rat in protocol 2. Data were resampled at 10 Hz. AVNS and CSP were perturbed according to binary white noise sequences. Both AVNS and CSP perturbation resulted in SNA and AP changes.

**Figure 4.**
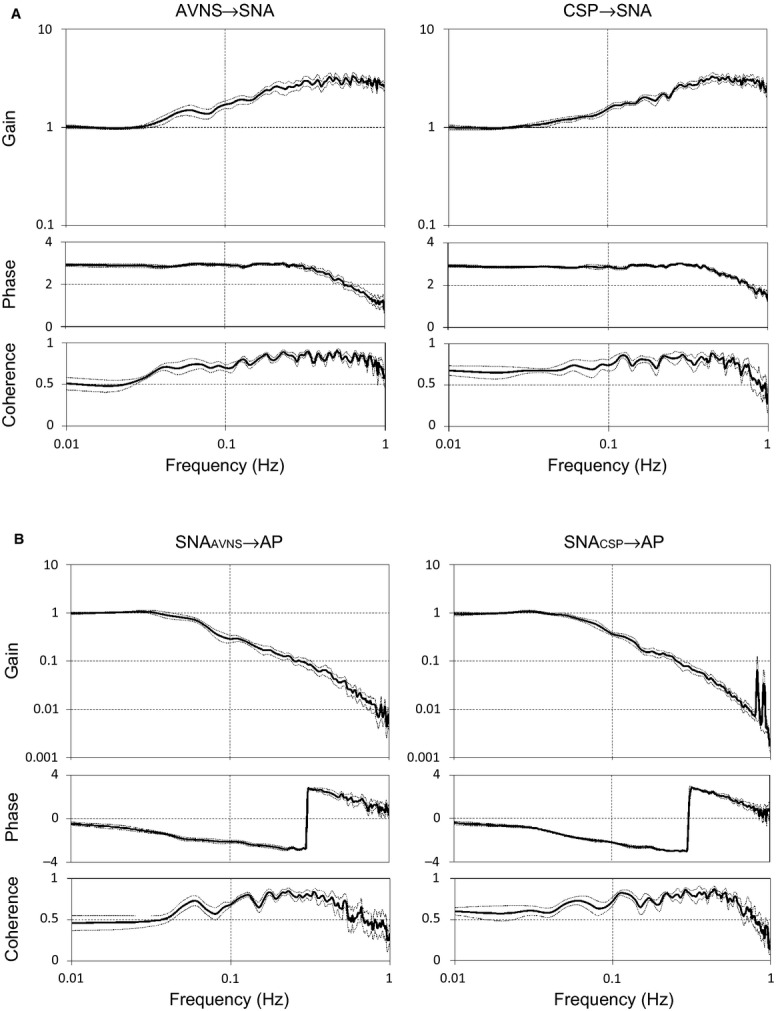
Dynamic transfer functions obtained under afferent vagal nerve stimulation or baroreflex control: transfer functions of AVNS to SNA (*left*) and baroreflex neural arc (CSP to SNA,* right*) (A); transfer functions of SNA changed by AVNS to AP (*left*) and baroreflex peripheral arc (SNA changed by CSP to AP,* right*) (B). Mean gain, phase (radian), and coherence are shown. The gain of transfer functions were normalized by the mean dynamic gain below 0.03 Hz in each data. Solid and dashed traces represent means ± SEM (*n* = 6). AVNS, afferent vagal nerve stimulation; SNA, sympathetic nerve activity; CSP, carotid sinus pressure; AP, arterial pressure.

Figure 3A shows typical time series of AVNS, SNA, and AP under AVNS perturbation. The duty cycle of AVNS was perturbed according to a binary white noise sequence. AVNS perturbation resulted in SNA and AP changes. Figure 3B shows typical time series of CSP, SNA, and AP under CSP perturbation. CSP was perturbed according to a binary white noise sequence (mean AP ± 20 mmHg). CSP perturbation resulted in SNA and AP changes via the carotid sinus baroreflex. Figure 4A shows the mean transfer functions of H_*AVNS‐SNA*_ (AVNS) and H_*CSP‐SNA*_ (baroreflex neural arc) obtained from 6 rats. Gain plots, phase plots, and coherence functions are shown. The gain increased as frequency increased for both H_*AVNS‐SNA*_ and H_*CSP‐SNA*_, indicating derivative characteristics. The phase approached –*π* radians at the lowest frequency, reflecting an out‐of‐phase relationship for both AVNS‐SNA and CSP‐SNA. The coherence function of AVNS‐SNA in the frequency range between 0.01 and 0.1 Hz was slightly lower than that of CSP‐SNA. Figure 4B shows the gain, phase, and coherence of the mean transfer functions of SNA*_AVNS_*‐AP and SNA*_CSP_*‐AP. The dynamic gain decreased as the input frequency increased for both AVNS and CSP, indicating low‐pass characteristics. The phase approached zero radians at the lowest frequency under both AVNS and CSP, reflecting that a rise in SNA increased AP. The coherence values did not differ between two groups. Table 1 summarize the gain and phase values of the transfer function of AVNS and carotid sinus baroreflex. The gain and phase at 0.01, 0.1, 0.5, and 0.75 Hz did not differ significantly between H_*AVNS→SNA*_ and H_*CSP→SNA*_, suggesting that the dynamic effect of AVNS on SNA resembled that of carotid baroreflex. Both the H_*SNA‐AP*_ obtained by perturbation of AVNS and CSP approximated a low‐pass filter, and the transfer functions (including the gain and phase) of AVNS and CSP matched well, indicating that AVNS induced changes in SNA to modulate AP in the same manner as carotid baroreflex (Table 2).

**Table 1.** Parameters of transfer functions of AVNS to SNA and baroreflex neural arc.

	AVNS (AVNS→SNA)	Baroreflex neural arc (CSP→SNA)
Gain		
0.01 Hz	1.02 ± 0.04	1.00 ± 0.05
0.1 Hz	1.71 ± 0.16	1.55 ± 0.08
0.5 Hz	3.18 ± 0.32	3.03 ± 0.25
0.75 Hz	2.94 ± 0.36	3.11 ± 0.28
Phase (radians)		
0.01 Hz	2.92 ± 0.16	2.93 ± 0.05
0.1 Hz	2.92 ± 0.06	2.90 ± 0.05
0.5 Hz	2.22 ± 0.15	2.49 ± 0.07
0.75 Hz	1.60 ± 0.16	1.99 ± 0.08

Data are expressed as means ± SEM. AVNS, afferent vagal nerve stimulation; SNA, sympathetic nerve activity; CSP, carotid sinus pressure; There are no significant differences between AVNS and baroreflex neural arc.

**Table 2.** Parameters of transfer functions of SNA changed by AVNS to AP and baroreflex peripheral arc.

	AVNS (SNA_avns_→AP)	Baroreflex peripheral arc (SNA_csp_→AP)
Gain		
0.01 Hz	0.97 ± 0.05	0.94 ± 0.07
0.1 Hz	0.29 ± 0.05	0.37 ± 0.03
0.5 Hz	0.04 ± 0.01	0.03 ± 0.01
0.75 Hz	0.013 ± 0.004	0.009 ± 0.002
Phase (radians)		
0.01 Hz	−0.46 ± 0.13	−0.40 ± 0.15
0.1 Hz	−2.13 ± 0.11	−2.20 ± 0.05
0.5 Hz	2.22 ± 0.23	2.07 ± 0.21
0.75 Hz	1.07 ± 0.37	1.12 ± 0.23

Data are expressed as means ± SEM. AVNS, afferent vagal nerve stimulation; SNA, sympathetic nerve activity; AP, arterial pressure; There are no significant differences between AVNS and baroreflex peripheral arc.

### Effect of AVNS on baroreflex function

**Figure 5.**
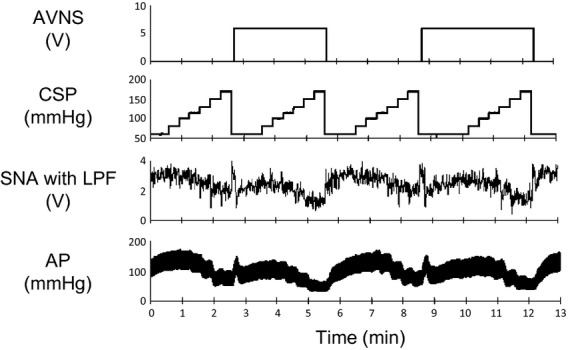
Typical time series of carotid sinus pressure (CSP), sympathetic nerve activity (SNA), and arterial pressure (AP) under baroreflex conditions (control) and with afferent vagal nerve stimulation (AVNS) from one rat in protocol 3. Data were resampled at 10 Hz. SNA and AP decreased in response to stepwise increments in CSP under both control and AVNS conditions. AVNS further decreased SNA and AP at each level of CSP. SNA with LPF, sympathetic nerve activity with low‐pass filter (cutoff frequency; 0.5 Hz).

Figure 5 shows recordings of CSP, SNA, and AP in the presence and absence of AVNS. The stepwise increase (20 sec/step, from 60 to 170 mmHg) in CSP decreased SNA and AP, while AVNS further decreased SNA and AP at each stepwise change in CSP.

**Figure 6.**
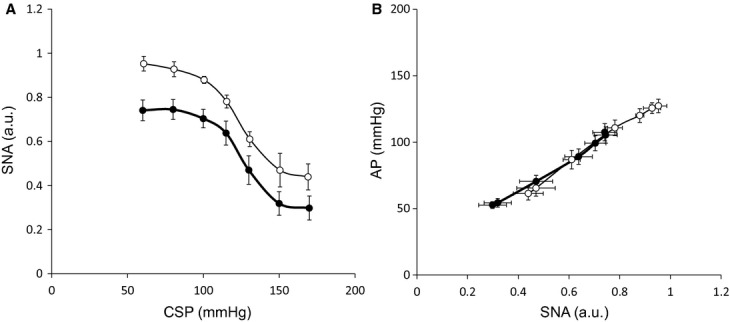
Mean baroreflex neural arc (A) and peripheral arc (B) under control condition (open circle) and with afferent vagal nerve stimulation (AVNS) (closed circle) in protocol 3. AVNS shifts the neural arc to lower SNA (A), but does not change the peripheral arc (B). Data are expressed as means ± SEM (*n* = 6). SNA, sympathetic nerve activity; CSP, carotid sinus pressure; AP, arterial pressure.

**Figure 7.**
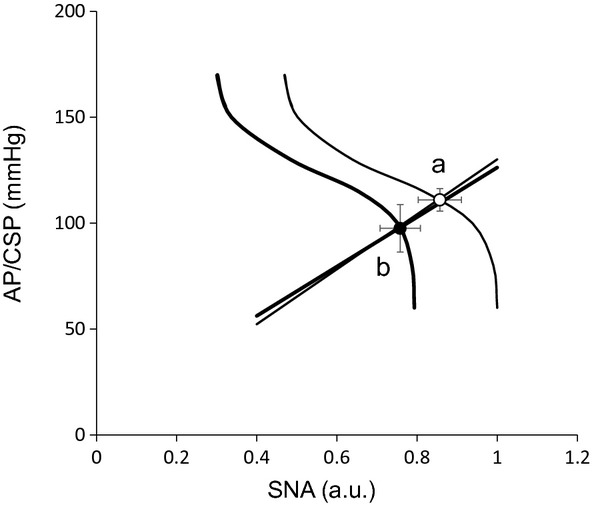
Baroreflex equilibrium diagram. The fine and bold solid lines indicate fitted logistic functions under control (open circle) and with afferent vagal nerve stimulation (AVNS) (closed circle) conditions, respectively. The operating point is determined from the intersection of the neural and peripheral arcs. The shift in neural arc reduces AP by 13.9 ± 8.8 mmHg and SNA by 0.12 ± 0.08 a.u. (from *point a* to *point b*) at the operating point.

Figure 6 shows the mean baroreflex neural and peripheral arcs in the presence and absence of AVNS obtained from 6 rats. The neural arc showed a sigmoidal relationship between CSP and SNA (Fig. 6A). In the neural arc, the response range of SNA (*P*_1_), the coefficient of gain (*P*_2_), midpoint of the operating range (*P*_3_), and maximum gain did not differ between the baroreflex control and AVNS. However, AVNS significantly reduced minimum SNA (*P*_4_) from 0.459 ± 0.148 to 0.295 ± 0.096, suggesting that AVNS caused a parallel downward shift of the baroreflex neural arc (Table 3). The peripheral arc showed a linear relationship between SNA and AP (Fig. 6B). In the peripheral arc, AVNS did not alter any of the baroreflex parameters (Table 3). The operating point determined from the intersection of the neural and peripheral arcs was shifted toward lower SNA and AP (from point *a* to *b*) by AVNS (Fig. 7).

**Table 3.** Effects of AVNS on the neural and peripheral arc parameters and operating point.

	Control (baroreflex)	AVNS
Operating point		
AP, mmHg	110.6 ± 5.3	96.7 ± 11.2*
SNA, a.u.	0.860 ± 0.024	0.745 ± 0.050*
Neural arc		
*P*_1_, a.u.	0.534 ± 0.153	0.499 ± 0.075
*P*_2_, a.u./mmHg	0.091 ± 0.012	0.095 ± 0.014
*P*_3_, mmHg	122.0 ± 5.3	124.8 ± 6.8
*P*_4_, a.u.	0.459 ± 0.148	0.295 ± 0.096*
Gmax, a.u./mmHg	−0.0124 ± 0.004	−0.012 ± 0.002
Peripheral arc		
a, mmHg/au	129.7 ± 35.1	116.7 ± 30.3
b, mmHg	0.4 ± 21.3	9.6 ± 17.3

Data are expressed as means ± SD. AVNS, afferent vagal nerve stimulation; AP, arterial pressure; SNA, sympathetic nerve activity; *P*1, response range of SNA; *P*2, coefficient of gain; *P*3, midpoint of the operating range; *P*4, minimum SNA; Gmax, maximum gain; a, slope; b, intercept.

* *P *<**0.05 versus control.

## Discussion

In the present study, we demonstrated the direct isolated effect of AVNS on baroreflex‐controlled SNA. The key new findings are as follows. (1) AVNS dose‐dependently inhibits SNA showing threshold and saturation, and H_*AVNS‐SNA*_ shows the characteristics of high‐pass filter resembling the baroreflex neural arc. (2) The two H_*SNA‐AP*_, including gain and phase, obtained by perturbation of AVNS (SNA_*AVNS*_–AP) and CSP (SNA_*CSP*_–AP) match well. (3) AVNS causes a parallel downward shift of the CSP–SNA relation (neural arc) without changing the maximal gain or operating range, but does not affect the SNA–AP relation (peripheral arc). These results suggest that AVNS resets the baroreflex neural arc and induces sympathoinhibition in the same manner as the native baroreflex.

### Electrical vagal afferent stimulation and sympathetic nervous systems

The vagal nerve consists mainly of A‐delta and C fibers, and 80–90% of the nerve fibers are afferent nerves (Schultz 2001). Approximately 75% of the afferent fibers are unmyelinated C fibers known to be involved in cardiovascular reflexes such as Bezold–Jarish reflex (Marmarelis and Marmarelis 1978) and cardiopulmonary reflex (Aviado and Schmidt 1995; Moore et al. 2004). Vagal afferent input is also known to deliver its signal to the nucleus tractus solitarius and modify not only respiration but also SNA (Clement et al. 1972; Aviado and Schmidt 1995; Moore et al. 2004). In protocol 1, we showed that AVNS dose‐dependently inhibits both SNA and AP. Verberne and Guyenet (1992) demonstrated that intravenous injection of phenylbiguanide that stimulates vagal afferent input inhibits barosensitive neurons in the rostral ventrolateral medulla in rats. In contrast to these previous studies, we kept CSP constant to avoid the buffering effect of baroreflex in the present study, because AP reduction induced by exogenous SNA inhibition increases SNA via baroreflex. Our method allowed us to evaluate the pure effect of AVNS on SNA, and we detected a maximum of 28.0 ± 10.3% reduction in SNA from the baseline level.

In protocol 2, we showed that the dynamic effects on both SNA and AP did not differ between AVNS and carotid baroreflex (Fig. 4A and B). The transfer function H_*AVNS‐SNA*_ has the characteristics of high‐pass filter, resembling the baroreflex neural arc. Ikeda et al. 1996 demonstrated that the dynamic transfer function of baroreflex neural arc represents high‐pass filter and that of peripheral arc low‐pass filter. It is conceivable that similar to baroreceptor afferent, AVNS inhibits SNA through the central nucleus tractus solitarius‐rostral ventrolateral medulla pathway. Previous studies indicated that the vagal afferent input and the arterial baroreflex might share common central pathways (Merahi et al. 1992; Pires et al. 1998). Importantly, both the AVNS‐induced and baroreflex‐induced changes in SNA showed almost the same characteristics of low‐pass filter in controlling AP. Our results strongly suggest that both AVNS and baroreflex afferent inputs integrate in the brain and affect cardiovascular system via SNA in a similar manner.

### Electrical vagal afferent stimulation and baroreflex function

Because the baroreflex is the most powerful regulator of SNA, we examined the impact of AVNS on the static baroreflex system in this study. The arterial baroreflex system is one of the most important negative‐feedback systems that stabilize AP against exogenous disturbances. When AP is decreased by exogenous perturbation such as blood loss, the decreased AP is sensed by arterial baroreceptors. The arterial baroreflex then increases SNA to buffer the reduction in AP. In such circumstances, SNA and AP change reciprocally. On the other hand, when SNA is changed by an exogenous perturbation such as emotional stress, SNA, and AP change in parallel. In protocol 3, we performed a baroreflex open‐loop experiment and identified the static characteristics of the neural and peripheral arcs over a wide operating range. As expected, AVNS shifted the neural arc toward lower SNA at all CSP levels (Fig. 6A). In contrast, AVNS had little effect on the peripheral arc (Fig. 6B). In other words, the AP responses to SNA changes were indistinguishable regardless of the absence or presence of AVNS. When we combined the neural and peripheral arcs to yield a baroreflex equilibrium diagram (Fig. 7), the closed‐loop operating point, determined from the intersection of the neural and peripheral arcs, shifted from point (a) to (b) during AVNS. Despite a significant shift in the closed‐loop operating point, there were no significant alterations in both the neural and peripheral arc gains at the operating point (Table 2). The fact that AVNS induced resetting of the baroreflex is consistent with the impact of other reflexes such as muscle mechanoreflex (Yamamoto et al. 2004) peripheral chemoreflex and central chemoreflex (Kara et al. 2003) on baroreflex function. The AVNS‐induced resetting of the arterial baroreflex neural arc may involve central interaction in the brain. These data suggest that AVNS‐evoked depolarization of vagal afferent C fibers may deliver the signal to the nucleus tractus solitaries and in turn, reset the baroreflex neural arc toward the direction of sympathoinhibition.

### Vagal nerve stimulation as a therapeutic device for heart failure

**Figure 8.**
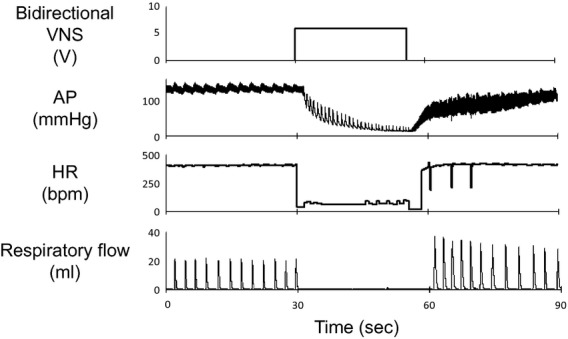
Time series of arterial pressure (AP), heart rate (HR), and respiratory flow under baseline condition (0–30 sec) and bidirectional vagal nerve stimulation from one rat. High‐amplitude (6 V) bidirectional vagal nerve stimulation markedly reduces heart rate by more than 100 beats/min accompanied by significant hypotension and apnea.

Many reports have indicated that VNS improves heart failure in animal models and humans (Li et al. 2004; De Ferrari et al. 2011). In this study, we fixed the frequency (20 Hz) and pulse width (0.2 msec) of ANVS as used by Li et al. in a chronic VNS study in CHF rats (Li et al. 2004), and varied the stimulation amplitude from 2 to 8 V to speculate how chronic VNS affects SNA. Our results indicate that VNS exhibits sympathoinhibition at 4 to 8 V but not at 2 V. Li et al. (2004) used a stimulation intensity of 0.1–0.13 mA, which is equivalent to stimulation amplitude of 2.5–3.5 V in our study (given the impedance of vagal nerves to be around 15 ± 3 kΩ). These findings thus suggest that the direct sympathoinhibitory effect of VNS was not significant in chronic VNS for heart failure. Moreover, in a preliminary experiment in which we conducted bidirectional VNS by electrically stimulating right vagal nerves in both afferent and efferent fibers in rats under spontaneous breathing condition, we found that bidirectional VNS at high amplitude (6–8 V) and 20 Hz markedly reduced heart rate by more than 100 beats/min and induced hypotension and apnea (Fig. 8). These phenomena induced by bidirectional VNS are consistent with previous reports in human and animals. Previous studies reported that 10–20% reduction in heart rate by vagal efferent stimulation did not affect pressure regulation via baroreflex (Kawada et al. 2011), whereas 50% reduction in heart rate by VNS caused significant hypotension (Shimizu et al. 2009). In addition, vagal afferent input also affects the respiratory center (Adrian 1933). In a clinical trial using CardioFit system, VNS was titrated by reduction in heart rate or development of intolerable effects including hoarseness and cough reflex, and up‐titration was limited by such adverse effects in 70% of the patients (De Ferrari et al. 2011). In an animal study, VNS improved heart failure in a dog model in which the amplitude of VNS was fixed at a level that did not reduce heart rate or cause any major adverse effects such as chronic coughing (Hamann et al. 2013). Considering these previous data, we speculate that low‐intensity VNS improves heart failure but does not reduce SNA directly. On the contrary, high‐intensity VNS reduces SNA directly, but may not be used in conscious animals because of severe bradycardia, hypotension, and respiratory effect. Further investigations are required to reduce SNA by electrical VNS without harming circulation or respiration, or causing other adverse effects.

### Limitations

There are several limitations in this study. First, as anesthesia affects the autonomic nervous system, the results might have been different without anesthesia. Second, we used normal rats in this study. Heart failure is known to alter the sensitivity of neural fibers and receptors that evoke physiological reflexes, which may vary the threshold of vagal fibers in response to electrical stimulation. In addition, we have only assessed the acute responses of SNA and baroreflex function against AVNS in this study. Thus, the present results cannot be directly extrapolated to chronic VNS therapy in rats or patients with heart failure. Third, isolation of the carotid sinus regions may stimulate carotid chemoreceptors. However, in determining baroreflex functions, this factor was present in trials with and without AVNS. Therefore, it is fair to say that this factor may not affect our conclusion of baroreflex resetting during AVNS.

## Conclusion

In conclusion, in anesthetized normal rats, AVNS resets the baroreflex neural arc and induces sympathoinhibition in the same manner as native carotid baroreflex, because AVNS causes a parallel shift of the CSP–SNA relation (neural arc) without changing the maximal gain or operating range, whereas AVNS has no effect on the SNA–AP relation (peripheral arc). Further studies are required to examine AVNS‐induced sympathoinhibition in the setting of chronic heart failure.

## Acknowledgments

The authors thank the staff of the Department of Cardiovascular Medicine for technical support.

## Conflict of Interest

The authors declare no conflicts of interest, financial or otherwise.

## References

[b1] Adrian E. D. 1933 Afferent impulses in the vagus and their effects on respiration. J. Physiol.; 79:332-358. 16994466 10.1113/jphysiol.1933.sp003053 PMC1394842

[b2] Aviado D. M. Jr Schmidt C. F. 1995 Reflexes from stretch receptors in blood vessels, heart and lungs. Physiol. Rev.; 35:247-300. 14384508 10.1152/physrev.1955.35.2.247

[b4] Clement D. L. Pelletier C. L. Shepherd J. T. 1972 Role of vagal afferents in the control of renal sympathetic nerve activity in the rabbit. Circ. Res.; 31:824-830. 4641779 10.1161/01.res.31.6.824

[b5] De Ferrari G. M. Crijns H. J. Borggrefe M. Milasinovic G. Smid J. Zabel M. 2011 Chronic vagus nerve stimulation: a new and promising therapeutic approach for chronic heart failure. Eur. Heart J.; 32:847-855. 21030409 10.1093/eurheartj/ehq391

[b6] Hamann J. J. Ruble S. B. Stolen C. Wang M. Gupta R. C. Rastogi S. 2013 Vagus nerve stimulation improves left ventricular function in a canine model of chronic heart failure. Eur. J. Heart Fail.; 15:1319-1326. 23883651 10.1093/eurjhf/hft118 PMC3895958

[b7] Ikeda Y. Kawada T. Sugimachi M. Kawaguchi O. Shishido T. Sato T. 1996 Neural arc of baroreflex optimizes dynamic pressure regulation in achieving both stability and quickness. Am. J. Physiol.; 271:H882-H890. 8853321 10.1152/ajpheart.1996.271.3.H882

[b8] Kamiya A. Kawada T. Shimizu S. Sugimachi M. 2011 Closed‐loop spontaneous baroreflex transfer function is inappropriate for system identification of neural arc but partly accurate for peripheral arc: predictability analysis. J. Physiol.; 589:1769-1790. 21486839 10.1113/jphysiol.2011.203455 PMC3099029

[b9] Kara T. Narkiewicz K. Somers V. K. 2003 Chemoreflexes–physiology and clinical implications. Acta Physiol. Scand.; 177:377-384. 12609009 10.1046/j.1365-201X.2003.01083.x

[b10] Kashihara K. Kawada K. Yanagiya Y. Uemura K. Inagaki M. Sugimachi M. 2003 Bezold‐Jarisch reflex attenuates dynamic gain of baroreflex neural arc. Am. J. Physiol.; 285:H833-H840. 10.1152/ajpheart.01082.2002 12714325

[b11] Kawada T. Sugimachi M. Sato T. Miyano H. Shishido T. Miyashita H. 1997 Closed‐loop identification of carotid sinus baroreflex open‐loop transfer characteristics in rabbits. Am. J. Physiol.; 273:H1024-H1031. 9277523 10.1152/ajpheart.1997.273.2.H1024

[b12] Kawada T. Shimizu S. Li M. Kamiya A. Uemura K. Sata Y. 2011 Contrasting effects of moderate vagal stimulation on heart rate and carotid sinus baroreflex‐mediated sympathetic arterial pressure regulation in rats. Life Sci.; 89:498-503. 21855552 10.1016/j.lfs.2011.07.026

[b13] Kent B. B. Drane J. W. Blumenstein B. Manning J. W. 1972 A mathematical model to assess changes in the baroreceptor reflex. Cardiology; 57:295-310. 4651782 10.1159/000169528

[b14] La Rovere M. T. Bigger J. T. Jr Marcus F. I. Mortara A. Schwartz P. J. ATRAMI (Autonomic Tone and Reflexes After Myocardial Infarction) investigators. 1998 Baroreflex sensitivity and heart‐rate variability in prediction of total cardiac mortality after myocardial infarction. Lancet; 351:478-484. 9482439 10.1016/s0140-6736(97)11144-8

[b15] Lechat P. Hulot J. S. Escolano S. Mallet A. Leizorovicz A. Werhlen‐Grandjean M. 2001 Heart rate and cardiac rhythm relationships with bisoprolol benefit in heart failure in CIBIS II trial. Circulation; 103:1428-1433. 11245648 10.1161/01.cir.103.10.1428

[b16] Levy M. N. Blattberg B. 1976 Effect of vagal stimulation on the overflow of norepinephrine into the coronary sinus during cardiac sympathetic nerve stimulation in the dog. Circ. Res.; 38:81-84. 1245024 10.1161/01.res.38.2.81

[b17] Li M. Zheng C. Sato T. Kawada T. Sugimachi N. Sunagawa K. 2004 Vagal nerve stimulation markedly improves long‐term survival after chronic heart failure in rats. Circulation; 109:120-124. 14662714 10.1161/01.CIR.0000105721.71640.DA

[b18] Marmarelis P. Z. Marmarelis V. Z. 1978 131-221 The white noise method in system identi?cation. Analysis of physiological systems New York Plenum

[b19] Merahi N. Orer H. S. Laporte A. M. Gozlan H. Hamon M. Laguzzi R. 1992 Baroreceptor reflex inhibition induced by the stimulation of serotonin 3 receptors in the nucleus tractus solitaries of the rat. Neuroscience; 46:91-100. 1350667 10.1016/0306-4522(92)90011-p

[b20] Moore J. P. Hainsworth R. Drinkhill M. J. 2004 Pulmonary arterial distension and vagal afferent nerve activity in anaesthetized dogs. J. Physiol.; 555:805-814. 14742726 10.1113/jphysiol.2003.057919 PMC1664861

[b21] Pires J. G. Silva S. R. Ramage A. G. Futuro‐Neto H. A. 1998 Evidence that 5‐HT3 receptors in the nucleus tractus solitaries and other brainstem areas modulate the vagal bradycardia evoked by activation of the von Bezold‐Jarisch reflex in the anesthetized rat. Brain Res.; 791:229-234. 9593908 10.1016/s0006-8993(98)00109-7

[b22] Sabbah H. N. Shimoyama H. Kono T. Gupta R. C. Sharov V. G. Scicli G. 1994 Effect of long‐term monotherapy with enalapril, metoprolol and digoxin on the progression of left ventricular dysfunction and dilation in dogs with reduced ejection fraction. Circulation; 89:2852-2859. 8205701 10.1161/01.cir.89.6.2852

[b23] Sato T. Kawada T. Inagaki M. Shishido T. Takaki H. Sugimachi M. 1999 New analytic framework for understanding sympathetic baroreflex control of arterial pressure. Am. J. Physiol.; 276:H2251-H2261. 10362709 10.1152/ajpheart.1999.276.6.H2251

[b24] Schultz H. D. 2001 Cardiac vagal chemosensory afferents. Function in pathophysiological states. Ann. NY Acad. Sci.; 940:59-73. 11458708

[b25] Schwaltz P. J. De Ferrari G. M. 2011 Sympathetic‐parasympathetic interaction in health and disease: abnormalities and relevance in heart failure. Heart Fail. Rev.; 16:101-107. 20577900 10.1007/s10741-010-9179-1

[b26] Schwartz P. J. Vanoli E. Stramba‐Badiale M. De Ferrari G. M. Billman G. E. Foreman R. D. 1998 Autonomic mechanisms and sudden death. New insights from analysis of baroreceptor reflexes in conscious dogs with and without myocardial infarction. Circulation; 78:969-979. 3168199 10.1161/01.cir.78.4.969

[b27] Shimizu S. Akiyama T. Kawada T. Shishido T. Yamazaki T. Kamiya A. 2009 In vivo direct monitoring of vagal acetylcholine release to the sinoatrial node. Auton. Neurosci.; 148:44-49. 19278905 10.1016/j.autneu.2009.02.006

[b28] Smith B. N. Dou P. Barber W. D. Dudek F. E. 1998 Vagally evoked synaptic currents in the immature rat nucleus tractus solitarii in an intact in vitro preparation. J. Physiol.; 512:149-162. 9729625 10.1111/j.1469-7793.1998.149bf.x PMC2231195

[b29] Tracey K. J. 2007 Physiology and immunology of the cholinergic anti‐inflammatory pathway. J. Clin. Invest.; 117:289-296. 17273548 10.1172/JCI30555 PMC1783813

[b30] Tsutsumi T. Ide T. Yamato M. Kudou W. Andou M. Hirooka Y. 2008 Modulation of the myocardial redox state by vagal nerve stimulation after experimental myocardial infarction. Cardiovasc. Res.; 77:713-721. 18065771 10.1093/cvr/cvm092

[b31] Vanoli E. De Ferrari G. M. Stramba‐Badiale M. Hull S. S. Jr Foreman R. D. Schwartz P. J. 1991 Vagal stimulation and prevention of sudden death in conscious dogs with a healed myocardial infarction. Circ. Res.; 68:1471-1481. 2019002 10.1161/01.res.68.5.1471

[b32] Verberne A. J. Guyenet P. G. 1992 Medullary pathway of the Bezold‐Jarisch reflex in the rat. Am. J. Physiol.; 263:R1195-R1202. 1481927 10.1152/ajpregu.1992.263.6.R1195

[b33] Yamamoto K. Kawada T. Kamiya A. Takaki H. Miyamoto T. Sugimachi M. 2004 Muscle mechanoreflex induces the pressor response by resetting the arterial baroreflex neural arc. Am. J. Physiol.; 286:H1382-H1388. 10.1152/ajpheart.00801.2003 14630630

